# Comparing 16 Different Dual–Tasking Paradigms in Individuals With Multiple Sclerosis and Healthy Controls: Working Memory Tasks Indicate Cognitive–Motor Interference

**DOI:** 10.3389/fneur.2020.00918

**Published:** 2020-08-28

**Authors:** Carmela Leone, Lousin Moumdjian, Francesco Patti, Ellen Vanzeir, Ilse Baert, Renee Veldkamp, Bart Van Wijmeersch, Peter Feys

**Affiliations:** ^1^Faculty of Rehabilitation Sciences, REVAL Rehabilitation Research Center, Hasselt University, Hasselt, Belgium; ^2^Faculty of Arts and Philosophy, IPEM Institute of Psychoacoustics and Electronic Music, Ghent University, Ghent, Belgium; ^3^Section of Neurosciences, Department of Medical Sciences, Surgical and Advanced Technologies G.F. Ingrassia, University of Catania, Catania, Italy; ^4^Rehabilitation and MS Centre Overpelt, Overpelt, Belgium; ^5^FBI, BIOMED, Faculty of Life Sciences and Physiotherapy, Hasselt University, Hasselt, Belgium

**Keywords:** cognitive–motor interference, discriminative complex walking task, dual-task cost, walking, working-memory

## Abstract

**Background:** Cognitive–motor interference (CMI) is measured by dual-tasking (DT), which involves motor and cognitive tasks. There is no consensus as to whether CMI is present in multiple sclerosis (MS).

**Objectives:** We investigated the effects of 16 DT conditions by measuring motor complexity, cognitive domain, and task difficulty.

**Method:** In total, 40 persons with MS (pwMSs) with Expanded Disease Status Scale (EDSS) 3.2 ± 1.7 and 31 age- and sex-matched healthy controls (HCs) completed 2 single walking, 8 single cognitive, and 2 complex walking tasks and 16 cognitive–motor DT. The main outcomes were mean values of gait velocity and the percentage change from single to DT (motor DT costs, mDTCs) and mean values of cognitive task accuracy and the percentage changes (cognitive DTC, cDTC).

**Results:** Two-way analyses of variance showed the main effect of cognitive task yielded an *F* ratio of *F*_(4, 268)_ = 72.35, *p* < 0.01, for mean gait velocity, and an *F* ratio of *F*_(4, 304)_ = 17.12, *p* < 0.001, for mDTC, indicating that the mean velocity was significantly lower and the mDTC significantly higher for DS_B (mean = 1.27, SD = 0.03, and mean = 13.52, SD = 1.28, respectively). The main effect of cognitive task yielded an *F* ratio of *F*_(4, 116)_ = 84.32, *p* < 0.001, with the lowest average accuracy for DS_B (mean = 43.95, SD = 3.33); no effect was found for cDTC. In pwMSs, the EDSS accounted for 28% (*F* = 13.65, *p* = 0.001) of variance in a model predicting the highest mDTC.

**Conclusions:** Overall, among different cognitive tasks added, the Digit Span backward was the most interfering cognitive task over gait velocity and accuracy. The effect was similar independently from the motor complexity and the group. PwMSs and HCs behaved in a similar manner at all motor complexity levels and during all cognitive task.

## Introduction

Gait is a conscious, goal-oriented process, which requires higher-level cognitive functions, such as executive functioning, attention, memory, and vision (1). The non-automatic nature of gait may be revealed with dual-task (DT) paradigms, which are one of the most common ways to gauge the interaction (or the coupling) between gait and cognition. The simultaneous performance of a cognitive task during walking may worsen or unmask gait impairments, also known as called cognitive–motor interference (CMI). Simultaneous performance of a cognitive and a motor task may lead to a percentage change in one or both tasks (1), and it is usually quantified as DT cost (DTC), which is the percentage change from the single-task (ST) to the DT performance (2). This DTC can be in positive or negative direction, indicating a detrimental or a facilitating effect on a given task, respectively. No percentage change likely means no interference (3). Functional community ambulation requires an ability to walk safely while performing different cognitive tasks such as talking, thinking, storing a sequence of numbers/words, or retrieving words from memory. Therefore, a reduced capacity for DT associated with walking may limit the performance of common daily activities.

Persons with multiple sclerosis (pwMSs) frequently present both cognitive and walking dysfunction, so it is reasonable to assume that they may have more difficulties in DT associated with walking when compared to healthy controls (HCs).

As such, walking while simultaneously performing a cognitive task (DT of walking) may be a new methodological approach for the evaluation of overall disability in pwMSs, which can account for the different levels of motor difficulties associated with MS progression. Difficulties in DT associated with walking received attention in the last few years in MS research. Initial reviews of studies investigating DT in pwMSs showed an overall reduced walking performance during DT in pwMSs indicating motor interference as the primary effect of CMI is motor interference (4, 5). However, in subsequent years, other research has shown no significant differences in DTC on gait performance between persons with and without MS (6–8). For example, the systematic review of Learmonth et al. (8) indicated there is a non-significant minimal difference in CMI between pwMSs and HCs, while the most recent systematic review from Postigo-Alonso et al. (9) concluded there was a significant difference in CMI in pwMSs, although this investigated studies related specifically to motor task–and cognitive task–related factors. Therefore, it seems that the presence of a significant DTC in pwMSs compared with HCs is dependent on the type of spatiotemporal gait parameter analyzed (velocity, cadence, double support, etc.), as well as the type of the cognitive task added. For example, Postigo-Alonso et al. (9) found that velocity is the most sensitive gait parameter to CMI, but it does not discriminate between pwMSs and HCs, whereas the verbal fluency task with cadence and/or double support and Serial Subtracting 7's with cadence were the proficient in discriminating CMI between pwMSs and HCs. However, it should be noted that they did not include from their analyses the study of Hamilton et al. (10), as Digit Span (DS) was administered only in a forward direction, excluding to report on significant data regarding DTC of walking during a working memory task. Postigo-Alonso et al. (9) investigated the sensitivity and specificity of the following tasks: Alternate Alphabet, Serial Subtracting 7's, Serial Subtracting 3's, and verbal fluency.

The existing research on CMI in MS has notable limitations. First, it has focused predominantly on simple motor tasks such as balance, standing, or simply forward walking, without the investigation of more complex mobility tasks, which are commonly performed in daily life, such as walking while holding something in the hands (i.e., a mug). Second, the majority of studies have investigated only one or a maximum of three cognitive tasks (11) in the same sample population, missing the opportunity to adequately investigate whether CMI is related to the type of cognitive domain involved (working memory, set shifting, inhibitor controls attention, processing speed, etc.). Moreover, to our knowledge, no studies have investigated the impact of difficulty level, when the same cognitive and/or motor task is maintained. Likely, using different cognitive domains and at least two difficulty levels for each domain could help in revealing whether the underlying mechanisms of CMI is a competition for overall attention resources (limited sharing-capacity model) (12) or a competition for information-processing neural pathways (bottleneck model) (13) or both. Lastly, only a few studies have evaluated the impact of DT on cognitive performance with (10) or without (14, 15) a measure of DTC according to the formula of Baddeley et al. (16). Measuring the cognitive DTC together with the motor DTC would mean the prioritization of one task over the other or splitting prioritization equally between the two tasks.

Beyond task-related factors, sample-related variables, such as mobility and cognitive outcome, may be important contributing factors for CMI. The correlation between CMI and disability level is not clearly established. While a previous study reported a greater DTC in pwMSs with greater disabilities, a revision revealed that DTCs were found irrespective of the disability level (5). With respect to cognition measured by neuropsychological assessment, its contribution to CMI has been poorly investigated. Few studies have performed a comprehensive neuropsychological assessment (17, 18), whereas others typically measure only a single cognitive domain or a generic cognitive assessment that is not specific for MS-related cognitive impairment (5).

It is well-known that the effect of DT on gait velocity is overall related to impairments in executive functions and attention (19, 20). A DT study in individuals with Parkinson disease found that only those with executive dysfunction had an association between higher DTC and increased risk of falling (21).

The primary aim of the current study was to investigate which cognitive task interferes most with walking performance [motor interference or motor DTC (mDTC)] and cognitive accuracy [cognitive interference or cognitive DTC (cDTC)] under either single motor (only walking) or dual motor [walking while carrying a mug named as complex walking task (CWT)] conditions, investigating whether difference exists between pwMSs and sex- and age-matched HCs. The DT combination that produced the highest motor interference in pwMSs was used as referral mDTC for subsequent analyses used to verify whether any physical or cognitive sample-related factors predict discriminative mDTC in pwMSs.

## Materials and Methods

### Participants

People diagnosed with clinically defined multiple sclerosis (MS) according to the McDonalds criteria of an Expanded Disease Status Scale (EDSS) score ranging from 0 to 6.0 (inclusive), which was stable since for at least 6 months, were enrolled in this study along with age-matched HCs. Exclusion criteria included history of systemic diseases that may influence motor performance at time of enrolment, disability not related to MS disease, any musculoskeletal medical history, an MS relapse occurring within 50 days prior to inclusion, and/or treatment with intravenous corticosteroid or oral corticosteroid within 30 days prior to inclusion, pregnancy, or being unable to comply with the requirements of the protocol. Demographic data collected were sex, age, height, weight, body mass index, and school level. Educational attainment was assessed by three categories according to the number of years in school: less than 12, between 12 and 15, and more than 15. Written informed consent was obtained from all participants. Recruitment was carried out through diverse Flemish MS centers, professional networks of physical therapists and neurologists, and the Flemish MS Society by distribution of leaflets. The study was approved by the medical ethical committees of the university and MS centers. The study was performed in accordance with the Declaration of Helsinki.

### Study Design

This was designed as an observational case–control study. Testing procedures were performed on 2 different testing days. Descriptive characteristics, physical and cognitive baseline tests (neuropsychological examination) were recorded on day 1. The ST and DT experimental protocol (see below) were performed on day 2.

### Experimental Protocol

**Figure 1:**
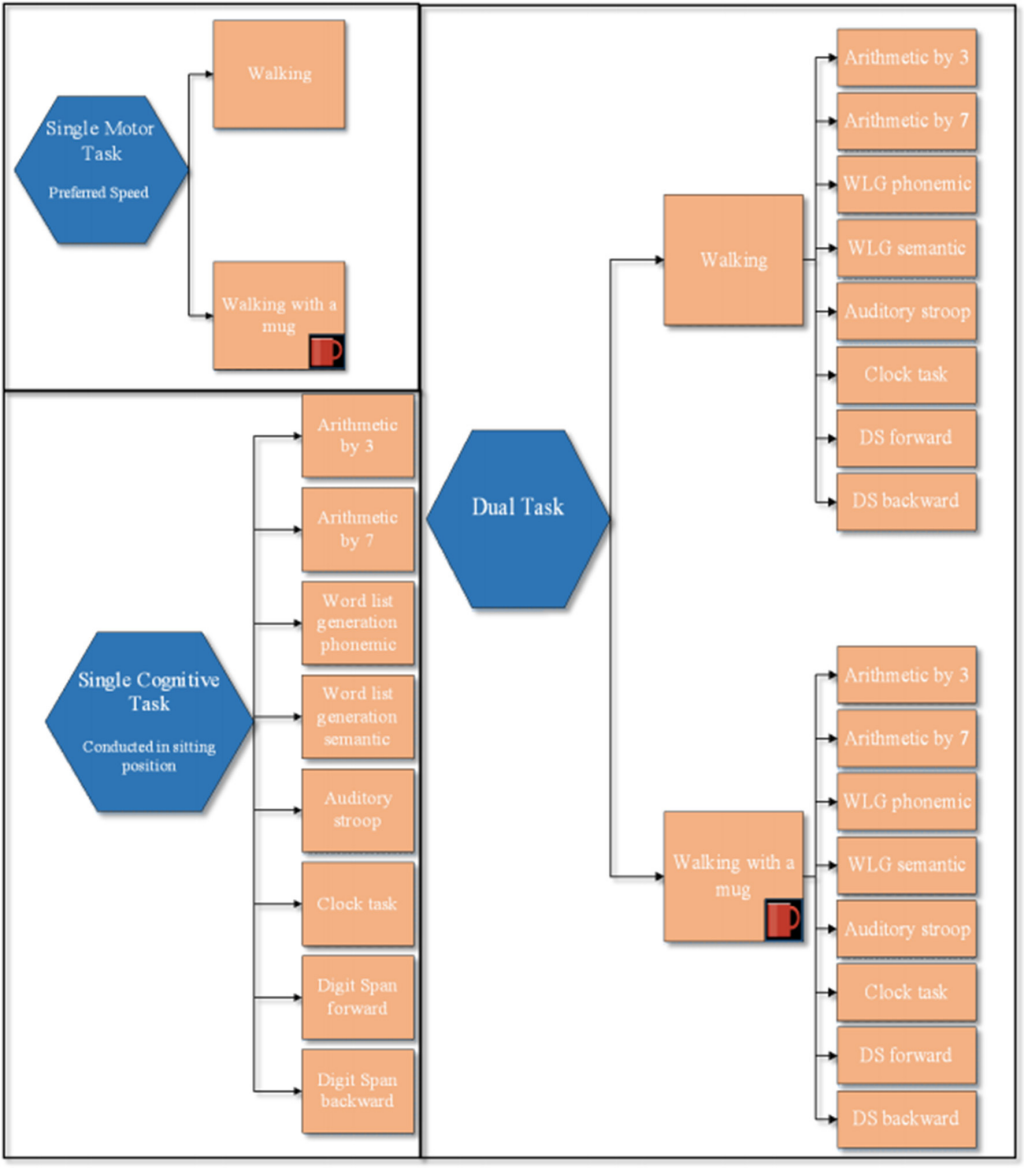
Experimental protocol. This figure illustrates: block of 2 single motor tasks, block of the 8 single cognitive tasks, and the two blocks of dual task paradigms: walking with and without a mug, while simultaneously performing the 8 cognitive tasks. The order of each ST within a block was randomized. The order in which tasks within each block were performed as the order between blocks was randomized.

As shown in Figure 1, all participants underwent all single motor and single cognitive tasks as well as each combination of single motor and single cognitive tasks (DTs) as reported below. Using a computer software, the order of each ST within a block was randomized. The order in which the blocks were performed was also randomized.

#### Single Motor Tasks

Within each block, trials of 15 and 60 s were conducted. Walking at their preferred velocity for 15 s (two trials) over an 8-m-long corridor was used as the referral ST for those DT using a cognitive task lasting 15 s, whereas walking at their preferred velocity for 60 s (one trial) was used as a referral for those DT using a cognitive task lasting 60 s. The CWT consisted of the previously explained motor ST, for 15 or 60 s, during which the participant was asked to carry a mug in their dominant hand. The mug was filled half way with water, and participants were asked not to spill the water during the entire experiment. Gait velocity (m/s) was recorded for all trials using APDM sensors. APDM's Mobility Lab™ (APDM Inc., http://apdm.com) is a portable gait and balance laboratory designed to streamline gait and balance assessment and to collect, store, analyze, and interpret data. Mobility Lab™ is composed of (1) a set wireless, body-worn Opal™ inertial sensors, each with a docking station, (2) an Access Point for wireless data transmission and submillisecond synchronization of the independent sensors, (3) user-friendly software to guide the user and subject(s) through the testing protocols, and (4) automated analysis and reporting of the recorded data.

#### Single Cognitive Tasks

With the exception of the DS forward (F) and backward (B), all tasks were conducted twice for a duration of 15 s, and an average was calculated. Both the DS_F and B were conducted once for a duration of 60 s; this test is longer in order to provide individuals the correct amount of time to listen (1 digit per second) and repeat the sequence of digits, as explained below. The number of digits was initially assessed by the administrator of neuropsychological assessment according to the individual's capacity (titrated DS).

Prior to trial initiation within each task, participants were instructed as to the content of each trial, which was followed by conducting a familiarization trial.

We grouped cognitive tasks according to the type of cognitive domain involved; in each group, two levels of task difficulty were applied:

**- Arithmetic tasks:** consisting of counting aloud backward (a) subtracting by 3 (subtracting 3's) and (b) subtracting by 7 (subtracting 7's) for 15 s; performance on the arithmetic tasks was measured by the total correct serial subtractions.

**- Inhibition of cognitive interference and attention tasks:** (a) Auditory Stroop (AS) (22) task: participants heard the words “high” and “low” spoken in either a high pitch (360 Hz) or a low pitch (180 Hz) for 15 s. They were instructed to indicate the pitch of the word they heard (ignoring the actual word presented) by responding verbally “high” or “low” as accurately and as quickly as possible. The stimulus was congruent when the word and pitch matched (e.g., “low” word spoken in low pitch) and incongruent when these did not (e.g., “low” word spoken in high pitch). The auditory stimuli were relayed to the participant via wireless earphones, and responses were collected via wireless headset microphones and recorded in the same program. Interstimuli interval was randomly delivered between 1.5 and 2 s. This was to avoid participants using the stimuli as a metronome for walking;

(b) “Clock task” (CT) (23): participants heard a time (e.g., 125) and were required to determine whether the two hands of the clock at the given time were in the same half (i.e., left or right) or opposite halves for 15 s. If the hands were the same half, they had to respond “yes;” if the hands were in opposite halves, they had to respond “no.” The stimuli were relayed and the answer collected as described for the AS. We assumed that this task was more difficult because it required greater cognitive processing. For AS and CT, we measured accuracy as percent of correct responses out of the amount of the given answers.

**- Working memory tasks:** Two-digit span (DS) tests measuring the short-term verbal memory were used. To perform DS (24), participants listened to a titrated string of digits (e.g., 3-2-5-7-9), at the presented rate of one per second (standard rate commonly used in neuropsychological tests as Wechsler Adult Intelligence Scales III and Memory Scales), and repeated them in (a) the same order (DS forward, DS_F) or (b) backward (DS_B). The digit sequence was delivered by an auditory program, and the participant responses were recorded by a wireless headset microphone. We calculated the percentage of digit sequences correctly recalled. The sequence length was assessed before the trial for each patient in order to determine the subject's DS (titrated): six trials were given at each sequence length starting from a 2-digit length; when five of six trials at a given length were correct, the length increased by one digit. Each participant's DS was determined as the last sequence length at which five out of six trials were correct.

**- Verbal fluency and executive functions:** Two forms of the modified Word List Generation (WLG) (25) were used: (a) the phonemic variant of WLG consisted of naming in 15 s as many words as possible starting with specific letters (randomly chosen among N, A, K, P, R, W), and (b) the semantic variant of WLG consisted of naming in 15 s as many words as possible belonging to a certain category (randomly chosen among animals, fruits and vegetables, or professions). For both of these tasks, we registered the number of words uttered in 15 s.

#### Cognitive–Motor DT Conditions

DT conditions consisted of simultaneously performing each of the above listed cognitive tasks during walking at preferred velocity with or without carrying a mug (CWT). The same number of trials and duration per trial was used as described for the ST conditions, and each DT block and conditions were randomized. During the experimental DT conditions, participants were instructed to simultaneously perform the motor and cognitive tasks. They were not given any instructions regarding prioritization of walking or cognitive task, but they were asked to perform both tasks as best as they could. Subjects paused for about 30–45 s between trials to allow time for the assessor to set up the next trial.

#### Calculating the Dual Task Cost (%)

The percentage DTCs were calculated for each DT combination performed. For this purpose, the DTC of each walking trial and of each cognitive task during the DT conditions was calculated according to the formula of Baddeley et al. (16) for each subject as follows:

$$\begin{array}{l} \text{ Motor DTC }=\left(\text{ST velocity }-\text{DT velocity}/\text{ST velocity}\right)*\;100\\ \text{Cognitive DTC }=\left(\text{ST accuracy }-\text{DT accuracy}/\text{ST accuracy}\right)*\;100 \end{array}$$

A positive DTC value indicated lower DT ability, whereas a negative value indicated higher DT ability.

### Sample-Related Contributing Factors

#### MS-Specific Data (Only for pwMSs)

Type of MS (relapsing remitting or progressive forms of MS), disease duration in years, and disability level as measured by the EDSS (26) were collected during baseline assessment.

### Data Collected for All Individual at Baseline Assessment

#### Motor Measures

The timed 25-ft walk test (T25WT) to evaluate short walking ability at the fastest velocity possible (27); the 6-min walking test (6MWT) to evaluate long distance walking ability at the fastest velocity possible for 6 min (28); the Timed Up and Go Test to evaluate dynamic balance (29); and the nine-hole peg test to evaluate fine motor skills and dexterity for each hand (30).

#### Cognitive Measures

The Brief Repeatable Battery of Rao incorporating tests of verbal memory acquisition and delayed recall (Selective Reminding Test); visual memory acquisition and delayed recall (10/36 Spatial Recall Test); attention, concentration, and velocity of information processing (Paced Auditory Serial Addition Test, Symbol Digit Modalities Test); and verbal fluency on semantic stimulus [Word List Generation (WLG)] (31); the Stroop Word Color Test used to assess executive functions (32); and the TMT-A and TMT-B to assess cognitive processing speed (TMT-A) and divided attention (TMT-B) (33). An abnormal score for each subtest was defined with the most stringent criterion of two standard deviations below the mean reported for healthy Dutch subjects. Subjects were classified as cognitively impaired if at least 3 tests had been failed (32, 34). According to the presence or absence of CI, pwMSs were subdivided into pwMSs with or without CI.

### Statistical Analysis

The Shapiro–Wilk test was used to assess normality in the continuous variables. Descriptive statistics are shown for pwMSs and HCs. Descriptive characteristics and clinical measures were compared between the two groups using an independent *t*-tests and χ^2^-tests (for discrete variables). The magnitude of the motor and cognitive DTCs was calculated for all participants.

Mean gait velocity and mean accuracy values as well as mDTC and cDTC values were each subjected to a two-way analysis of variance having one between-subjects factor (groups: pwMSs and HCs) and two within-subject independent variables: motor complexity (walking and CWT) and cognitive task (AS, CT, by3, by7, DS_F, DS_B, WLGph, WLGsem).

A Spearman or Pearson test was used for correlation analysis based on sample distribution. A multistep regression analysis (forward-stepwise selection) was performed to predict the highest mDTC. The probability of the *F*-value for variables in the model was set at.05, whereas the probability of *F*-value for the removal of variables was set at.10. All statistical tests were used with a two-tailed analysis and.05 as a level of significance. The statistical software SPSS version 20 was used for all analyses (IBM SPSS Statistics 20, ©IBM, Armonk, NY, USA).

## Results

### Demographic and Clinical Characteristics of the Two Groups

Forty pwMSs and 31 age-matched HCs participated in the study. Table 1 shows the demographic and clinical characteristics of the two groups. There were no significant differences for any of the variables examined (see inclusion criteria). The mean EDSS in the pwMSs group was 3.22 ± 1.73; the average disease duration was 12.45 ± 9.54 years.

**Table 1:** Demographic, physical, and cognitive baseline characteristics of pwMSs and HCs.

	**PwMSs (*n* = 40)**	**HCs (*n* = 31)**	***p***
Age (years)	47.7 ± 11.1	47.2 ± 13.8	n.s.
Female/male (%)	26/12 (70)	21/10 (67.7)	n.s.
BMI	24.4 ± 4.4	24.6 ± 3.9	n.s.
Education (years)	11.0 ± 0.6	12.7 ± 0.5	n.s.
Disease duration (years)	12.4 ± 9.5	–	n.s.
EDSS (mean ± SD)	3.2 ± 1.7	–	–
NHPT_DH	20.4 ± 5.3	18.0 ± 2.6	<0.05
NHPT_NDH	21.6 ± 5.9	18.6 ± 2.3	<0.05
TUG-3 meters (s)	8.8 ± 3.6	6.7 ± 1.1	<0.05
6MWT (m)	468.2 ± 135.1	599.9 ± 77	<0.001
T25WT (s)	5.2 ± 1.8	3.9 ± 0.5	0.001
CI (yes/no) (%)	11/29 (27.5)	0	<0.05
SRT-CLTR	30.1 ± 14.6	35.9 ± 18.0	n.s.
SRT-LTS	34.9 ± 12.4	39.0 ± 15.0	n.s.
SPART trial 1–5	31.3 ± 6.2	32.5 ± 4.1	n.s.
SPART trial B	3.8 ± 1.7	4.4 ± 2.0	n.s.
SPART trial 6	5.2 ± 2.3	6.4 ± 1.2	<0.05
SPART delayed	5.5 ± 2.0	6.5 ± 1.2	<0.05
SDMT	57.9 ± 15.5	71.7 ± 14.2	0.001
PASAT 3″	40.0 ± 11.9	46.5 ± 10.4	<0.05
WLG	30.5 ± 12.7	38.0 ± 18.0	<0.05
ST words	38.1 ± 11.1	48.7 ± 10.2	<0.001
ST colors	42.0 ± 11.6	55.4 ± 11.4	<0.001
ST interference w/c	47.8 ± 13.1	56.6 ± 10.2	<0.05
TMT A (s)	29.8 ± 17.3	25.9 ± 14.6	<0.05
TMT B (s)	64.6 ± 37.1	47.5 ± 22.0	<0.05

*Data are mean ± standard deviation*.

*pwMSs, persons with multiple sclerosis; HCs, healthy control; BMI, body mass index; EDSS, Expanded Disease Status Scale; NHPT, nine-hole peg test; DH, dominant hand; NDH, non-DH; TUG, Timed Up and Go Test; 6MWT, 6-min walking test; 25WT, time 25-ft walk test; CI, cognitive impairment; SRT, selective reminding test; CLTR, consistent long-term retrieval; LTS, long-term storage; n.s., not statistically significant; SPART, Spatial Recall Test; SDMT, Symbol Digit Modality Test; w/c, words/colors; PASAT, Paced Auditory Serial Addition Test; WLG, Word List Generation; ST, Stroop Test; TMT, Trail Making test*.

As reported in Table 1, the frequency of CI was significantly different between the two groups, with a frequency of 27.5% in the pwMSs compared with no cases in the HC groups (*p* < 0.05). There were significant differences between the two groups for many of the cognitive tests (for exceptions, see Table 1). As reported in Table 1, all motor variables were significantly different between the two groups.

### The Effects of 16 Different DT Paradigms on Walking Performance

#### Gait Velocity

**Table 3** shows average mean and SD of gait velocity in the two groups. The mean gait velocity during DT in pwMSs varied from 1.15 ± 0.29 m/s for CWT + DS_B to 1.27 ± 0.30 m/s for W+AS.

Between-subjects analysis yielded a small main effect of group, with an *F* ratio of *F*_(1, 67)_ = 15.23, *p* < 0.01, η^2^ = 0.18, such that the average gait velocity was significantly higher for HCs (mean = 1.44, SD = 0.04) than for PwMSs (mean = 1.22, SD = 0.04). The main effect of motor complexity yielded an *F* ratio of *F*_(1, 67)_ = 13.29, *p* < 0.01, η^2^ = 0.16, indicating that the mean velocity was significantly lower for CWT (mean = 1.32, SD = 0.03) than for DT with only walking (mean = 1.34, SD = 0.03). The main effect of cognitive task yielded an *F* ratio of *F*_(4, 268)_ = 72.35, *p* < 0.01, η^2^ = 0.52, indicating that the mean velocity was significantly lower for DS_B (mean = 1.27, SD = 0.03) and higher for AS (mean = 1.37, SD = 0.03), meaning that the DS_B led to a higher magnitude of gait velocity decrease.

**Figure 2:**
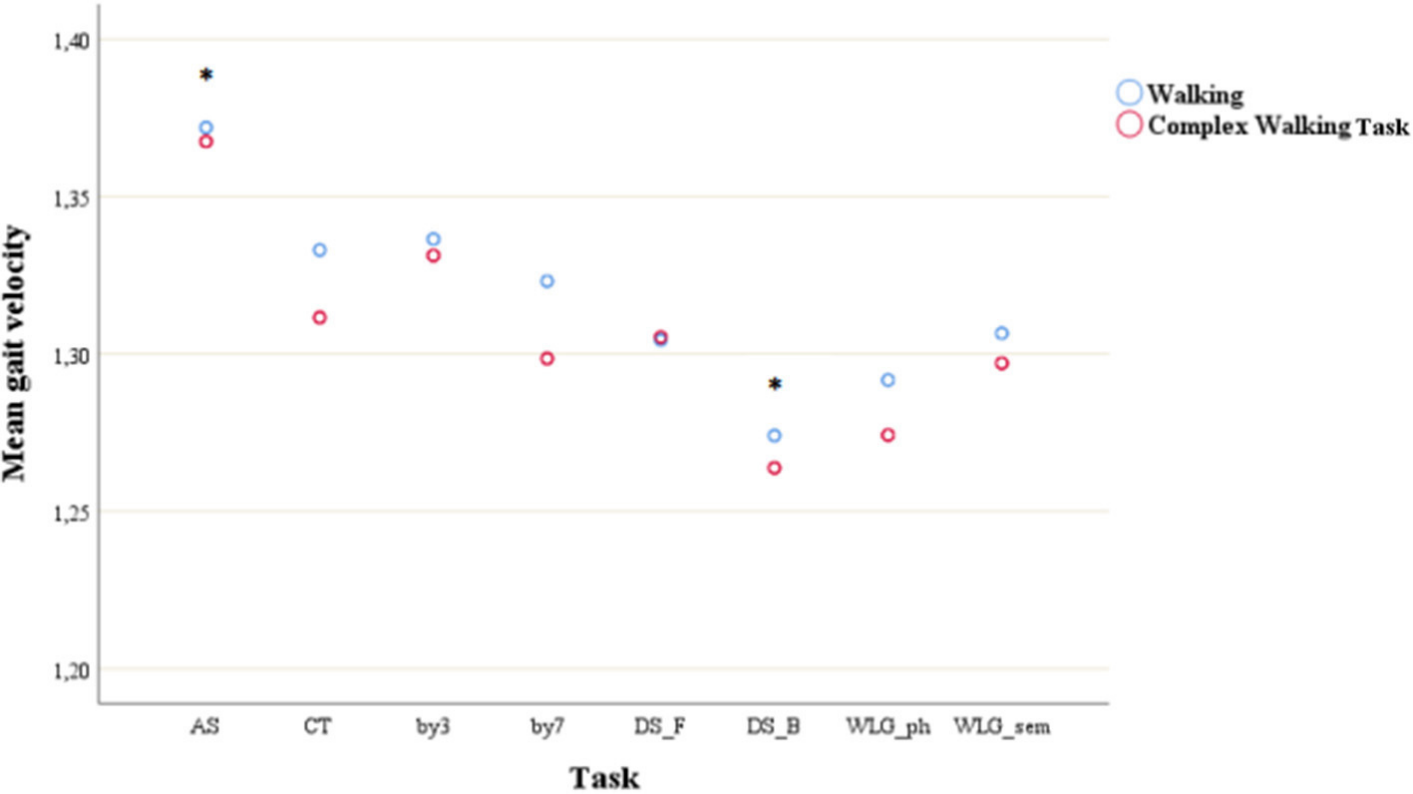
Mean gait velocity during simple walking and complex walking task across cognitive tasks. *post-hoc test showed significant difference with all the other tasks. AS, auditory stroop; CT, clock test; by3, subtracting by3; by7, subtracting by7; DS_F, digit span forwards; DS_B, digit span bacwards; WLG_ph, word list generation phonemic; WLG_sem, world list generation semantic.

As shown in Figure 2, *post-hoc* tests found that the mean gait velocity during AS were significantly different (all *p* < 0.01) with all the other mean gait velocities, meaning that AS had the lowest impact on velocity (mean = 1.37, SD = 0.03); mean gait velocity during DS_B was significantly different with all the other tasks, meaning that DS had the highest detrimental effect on velocity (mean = 1.27, *p* = 0.03).

The interaction effect between group ^*^ motor complexity, group ^*^ cognitive task, and group ^*^ motor complexity ^*^ cognitive task was non-significant, indicating that the effects of cognitive task and motor complexity were similar in the two groups; there was no significant interaction between motor complexity ^*^ cognitive task, meaning that motor complexity has similar effects on gait velocity among different cognitive tasks. Velocity changes in a similar manner over the groups and motor complexities.

#### Gait Velocity DTC

Table 2 reports mean motor DTC in the two groups. The mDTC in pwMSs varied from 8.81 ± 10.25 for the CWT + AS to 15.47 ± 9.96 for the DT combination of CWT + DS_B. Between-subjects analysis yielded no effect for group. The main effect of motor complexity yielded an *F* ratio of *F*_(1, 67)_ = 10.12, *p* = 0.002, η^2^ = 0.13, indicating that, although with a low effect size, the mean DTC was significantly lower for W (mean = 10.82, SD = 1.04) than for CWT (mean = 11.56, SD = 1.09).

**Table 2:** Mean gait velocity (m/s) and relative DTC (percentage) during all the single motor and cognitive-motor DT conditions (mean ± SD) presented for both pwMSs and HCs.

	**Walking velocity (m/s)**		**Motor DTC (%)**	
	**PwMSs**	**HCs**	**PwMS**	**HCs**
W + by3	1.22 ± 0.29	1.45 ± 0.13	11.91 ± 9.61	8.10 ± 7.87
W + by7	1.21 ± 0.18	1.43 ± 0.15	12.61 ± 0.28	9.43 ± 8.14
W + AS	1.26 ± 0.29	1.48 ± 0.13	9.16 ± 8.94	6.11 ± 6.69
W + CT	1.23 ± 0.29	1.44 ± 0.15	11.88 ± 10.35	8.84 ± 8.14
W + DS_F	1.19 ± 0.31	1.41 ± 0.15	12.26 ± 10.11	8.47 ± 8.50
W + DS_B	1.16 ± 0.31	1.38 ± 0.15	14.01 ± 11.23	10.97 ± 7.90
W + WLGph	1.18 ± 0.28	1.39 ± 0.14	14.81 ± 12.11	11.25 ± 8.84
W + WLGsem	1.20 ± 0.28	1.41 ± 0.13	13.24 ± 11.97	10.45 ± 8.05
CWT + by3	1.24 ± 0.30	1.43 ± 0.13	11.15 ± 10.87	9.53 ± 7.54
CWT + by7	1.19 ± 0.30	1.39 ± 0.14	14.07 ± 11.83	11.21 ± 8.65
CWT + AS	1.26 ± 0.29	1.47 ± 0.13	8.81 ± 10.25	6.83 ± 7.07
CWT + CT	1.19 ± 0.29	1.42 ± 0.14	13.79 ± 11.25	9.58 ± 8.52
CWT + DS_F	1.18 ± 0.29	1.42 ± 0.12	12.41 ± 9.43	7.82 ± 7.28
CWT + DS_B	1.15 ± 0.29	1.38 ± 0.15	15.47 ± 9.96	10.87 ± 8.44
CWT + WLGph	1.17 ± 0.28	1.38 ± 0.13	15.49 ± 12.31	12.58 ± 8.67
CWT + WLGsem	1.19 ± 0.29	1.40 ± 0.14	14.37 ± 12.73	10.97 ± 8.29

*DTC, dual-task cost; pwMSs, persons with multiple sclerosis; HCs, healthy control; w, walking; by3, counting backward by 3; by7, counting backward by 7; AS, Auditory Stroop; CT, Clock Test; DS, Digit Span; F, forwards; B, backward; WLG, word List Generation; ph, phonemic; sem, semantic; CWT, complex walking task*.

The main effect of cognitive task yielded an *F* ratio of *F*_(4, 304)_ = 17.12, *p* < 0.001, η^2^ = 0.20, indicating that the mean velocity's DTC was significantly lower for AS (mean = 7.73, SD = 0.98) and higher for WLG_ph and DS_B (mean = 12.83, SD = 1.12; mean = 13.53, SD = 1.28, respectively). *Post-hoc* tests showed significant differences between DTC of AS and all the other DTCs, meaning that AS has the lowest impact on velocity.

The interaction effect between cognitive task ^*^ group, motor complexity ^*^ group, cognitive task ^*^ motor complexity, and group ^*^ motor complexity ^*^ cognitive task was non-significant, indicating that the effects of cognitive task and motor complexity were similar in the two groups.

### The Effects of 16 Different DT Paradigms on Cognitive Performance

#### Mean Cognitive Task Accuracy

As shown in Table 3, the mean accuracy during DT in pwMSs varied from 38.93 ± 24.96 for W + DS_B to 98.80 ± 4.6 for CWT + by3.

**Table 3:** Mean accuracy of cognitive tasks and respective DTCs (percentage) during all the single cognitive and cognitive-motor DT conditions (mean ± SD) presented for both pwMSs and HCs.

	**Accuracy**		**Cognitive DTC (%)**	
**Task**	**PwMSs**	**HCs**	**PwMSs**	**HCs**
By3 sitt	98.05 ± 6.4	97.63 ± 8.21	n/a	
W + By3	95.58 ± 9.7	95.1 ± 14.77	2.32 ± 9.76	2.41 ± 14.02
CWT + By3	98.80 ± 4.6	97.45 ± 7.03	1.05 ± 7.82	−6.9 ± 23.33
By7 sitt	88.78 ± 18.33	86.47 ± 23.29	n/a	
W + By7	84.01 ± 23.62	93.38 ± 11.06	−10.17 ± 91.73	−29.18 ± 85.52
CWT + By7	90.61 ± 16.28	89.27 ± 17.28	−16.14 ± 88.37	−17.21 ± 57.01
AS sitt	91.89 ± 18.68	96.92 ± 7.88	n/a	
W + AS	96.14 ± 12.39	96.92 ± 8.84	−12.06 ± 37.79	−0.42 ± 10.51
CWT + AS	97.11 ± 10.37	98.97 ± 5.23	−15.02 ± 51.7	−2.53 ± 7.01
CT sitt	82.28 ± 20.12	84.74 ± 19.00	n/a	
W + CT	82.63 ± 20.49	87.69 ± 17.28	−5.45 ± 35.51	−7.44 ± 26.96
CWT + CT	81.93 ± 22.23	84.87 ± 20.14	−4.8 ± 39.78	−1.54 ± 21.08
DS_F sitt	72.13 ± 24.68	73.94 ± 19.17	n/a	
W + DS_F	69.49 ± 28.79	73.63 ± 24.08	−4.54 ± 80.49	−11.81 ± 41.04
CWT + DS_F	73.01 ± 24.83	65.62 ± 24.46	−7.91 ± 90.76	10.6 ± 53.03
DS_B sitt	47.43 ± 21.19	40.85 ± 27.29	n/a	
W + DS_B	38.9 ± 24.96	32.44 ± 24.39	13.43 ± 65.87	20.23 ± 64.07
CWT + DS_B	39.29 ± 28.06	46.26 ± 26.78	5.77 ± 67.36	−14.94 ± 78.82
WLGph sitt	90.48 ± 15.17	92.16 ± 11.99	n/a	
W + WLGph	92.04 ± 12.68	90.52 ± 16.34	−5.96 ± 31.02	−1.12 ± 24.29
CWT + WLGph	91.02 ± 11.78	95.12 ± 7.77	−6 ± 34.99	−5.41 ± 19.25
WLGsem sitt	99.21 ± 2.43	96.37 ± 10.56	n/a	
W + WLGsem	92.93 ± 14.12	96.44 ± 8.34	6.25 ± 14.48	−1.64 ± 20.16
CWT + WLGsem	96.93 ± 7.17	98.08 ± 4.32	2.22 ± 7.59	−3.79 ± 20.58

*DTC, dual-task cost; pwMSs, persons with multiple sclerosis; HCs, healthy controls; by3, counting backward by 3; SITT, sitting; W, walking; CWT, complex walking task; by7, counting backward by 7; AS, Auditory Stroop; CT, clock test; DS, Digit Span; F, forwards; B, backward; WLG, Word List Generation; ph, phonemic; sem, semantic*.

### Between-Subjects Analysis Yielded No Effect for Group

There was no significant effect of motor complexity on accuracy.

The main effect of cognitive task yielded an *F* ratio of *F*_(4, 116)_ = 84.32, *p* < 0.001, η^2^ = 0.74, with the lowest average accuracy for DS_B (mean = 43.95, SD = 3.33) and the highest for by3 (mean = 98.59, SD = 0.60), meaning that the DS_B led to a higher magnitude of accuracy's decrease.

**Figure 3:**
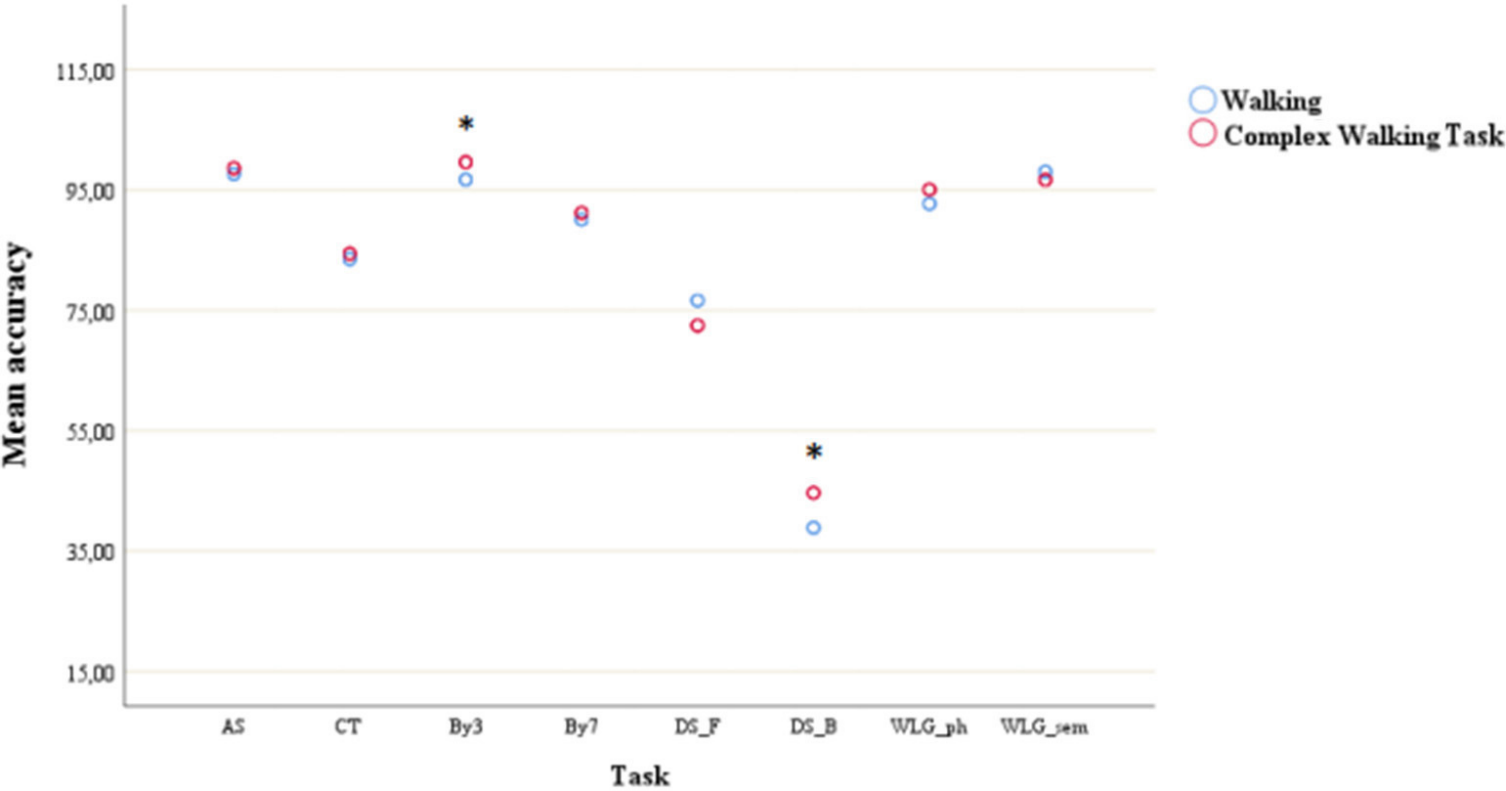
Mean accuracy during simple walking and complex walking task across cognitive tasks. *post-hoc test showed significant difference with all the other tasks. AS, auditory stroop; CT, clock test; by3, subtracting by3; by7, subtracting by7; DS_F, digit span forwards; DS_B, digit span bacwards; WLG_ph, word list generation phonemic; WLG_sem, world list generation semantic.

As shown in Figure 3, *post-hoc* tests showed that accuracy of DS_B was the only one significantly different (all *p* < 0.01) from all the other task accuracy.

The interaction effect between group ^*^ motor complexity, group ^*^ cognitive task, and group ^*^ motor complexity ^*^ cognitive task was non-significant, indicating that the main effects of task were similar in the two groups, as well as there were no significant interactions between motor complexity ^*^ task, meaning that motor complexity has similar effects on gait velocity among different cognitive tasks. Accuracy changes in the same manner over the groups and motor complexities.

#### Cognitive DTC

Regarding cDTC, it varied from −16.14 ± 88.37 for CWT + by7 to 13.43 ± 65.87 for W + DS in pwMSs group, and from −29.18 ± 85.52 for W + by7 to 20.23 ± 64.07 for W + DS_B in HC group. All cDTCs were associated with a high SD, and the HC group did not always show a better performance than pwMSs.

### Between-Subjects Analysis Yielded No Effect for Group

Within-subjects analysis yielded no main effect of motor complexity, neither of cognitive task, meaning that DTC scores were similar between the two groups, and percentage changes were similar across different cognitive tasks and motor complexity.

### Motor and Cognitive Baseline Outcomes as Contributing Factors of mDTC (CWT + DS_B) in pwMSs

The correlation analysis found moderate correlation, with “*r*” values ranging from 0.40 to 0.60 (*p* < 0.001), between the highest mDTC and many of the motor outcomes measured at baseline (Table 4). We found moderate correlations only with the presence of CI and TMT (forms A and B) among all the baseline cognitive measures. All the significant correlation values for the mDTC DS_B and CWT are listed in Table 4. A regression analysis showed that EDSS accounted for 28% (*F* = 13.65, *p* = 0.001) of the variance in the model predicting the mDTC of CWT + DS_B.

**Table 4:** Correlation analysis between discriminative DTC (CWT + DS_B) and physical/cognitive baseline characteristics.

**Participants' baseline characteristics**	**All sample**	**PwMSs**
**Discriminative DTC**		
NHPT-DH	0.35	0.46
TUG 3m	0.31	0.38
T25WT	0.48	0.56
6MWT	−0.41	−0.39
EDSS	–	0.52
CI	0.49	0.55
TMT A	0.36	0.51
TMT B	0.36	0.38

*DTC, dual-task cost; DS, digit Span; B, backward; CWT, complex walking task; NHPT DH, nine-hole peg test dominant hand; TUG, Timed Up and Go Test; T25WT, time 25-ft walk test; 6MWT, 6-min walking test; EDSS, Expanded Disease Status Scale; CI, cognitive impairment; TMT, Trail Making Test*.

## Discussion

### The Effects of 16 Different DT Paradigms on Walking Performance

First, our study confirms the well-known low gait velocity of PwMSs compared with HCs; we found that the gait velocity was not only different at the baseline but also during all the DT combinations. However, this difference was independently from the type of the cognitive secondary added task as well as from the complexity of motor task. Performing a complex motor task (walking while carrying a mug in our study design) did not interfere with the type of cognitive task, which means all the cognitive tasks were subjected to a decrease in gait velocity in pwMSs as well as in HCs. Those findings mean that velocity changes in a similar manner over the groups and motor complexities, which means both groups behave similarly when subjected to a DT paradigm. Participants independent from their group showed a significant decrease in gait velocity during DT whenever a cognitive distractor was added to single walking or to a CWT, although during a CWT the velocity resulted significantly lower than during DT combination with simple walking.

These findings are in line with previous research findings as described in several recent reviews (5, 35, 36). Al-Yahya et al. (35) systematically reviewed experimental studies measuring gait performance with and without a concurrent cognitive task. They found that cognitive tasks involving internal interfering factors (mental tracking task, working memory task, or verbal fluency) likely disturbed gait more than those involving external interfering factors (e.g., reaction time). In our study, *post-hoc* tests showed however that AS (a task involving external interfering factors) was the cognitive task with the lowest impact over both gait velocity and mDTC, whereas DS_B was the task with the highest interfering impact over gait velocity and mDTC. Similar to our results, the meta-analysis of Al-Yahya et al. (35) showed that there was an overall effect of adding a cognitive task on gait velocity in participants with neurological disorders, as well as in the HC group. Smith et al. (36) showed that both mental-tracking and verbal-fluency tasks resulted in a significant reduction in gait velocity in community-dwelling older adults with no significant pathology affecting gait. In pwMSs, Leone et al. (5) showed that the principal effect of DT performance on walking in MS was a reduction in gait velocity, followed by other effects on step length, support time, and cadence.

Regarding our aim to find whether a specific DT combination would be able to discriminate between groups, we found that no DT paradigm was able to differentiate pwMSs from HCs. The within-subject analysis revealed that the combination of CWT + DS_B was the highest one with significant difference with all the other DT combinations. The lack of significant difference between groups for CWT + DS_B is maybe due to the small sample size of our population.

Our results are mainly consistent with those found by Learmonth et al. (8). We did not find differences between pwMSs and HS for any of the DTCs investigated; no DT combinations significantly discriminate between people with and without MS as reported by Learmonth et al. (37).

On the other hand, our results are comparable to the results noted in another recent meta-analysis of studies that assess CMI in pwMSs vs. HCs provided by Postigo-Alonso et al. (9). It was reported that the DTC of gait velocity was significantly different in one study that used the DS as a cognitive distractor, as was done in this study (10), and that DTC of gait velocity was not different between pwMSs and the HC group in those studies involving walking and counting excluding 3's (11) or the alternate alphabet task (37, 38).

This common finding might be because we used DS as cognitive distractor, which is a working memory task, whereas the other studies used mental tracking tasks, such as Alternate Alphabet. Moreover, DS is considered itself a well-recognized neuropsychological test of working memory (39), which is known to be commonly impaired in pwMSs. However, this finding is in contrast with findings reported by Al-Yahya et al. (35), who showed that the cognitive tasks that interfered the most with gait velocity were mental tracking tasks. We did not find that these cognitive distractors gave the highest impact on walking/cognitive task; neither that they were able to discriminate between groups when we compared DTC. These conflicting results may also be explained by the duration of DT, as DS required participants to perform 60 s of a working memory task during walking, enabling them to perform 7–14 repetitions of an 8-m circuit. Therefore, 60 s of working memory, independently from the simultaneous walking during the mug carrying test, seems to be the perfect combination to evaluate CMI in pwMSs as well in HCs. The fact that no one of all the DTs we proposed, which covered almost all kind of cognitive domains, was significantly different between pwMSs, and the HC group should be considered relevant for two reasons. First, pwMSs already perform worse without distractors (gait velocity was lower than the HC group already in ST) so that an extra reduction of gait velocity would be negatively relevant, although not statistically significant in this study. Second, pwMSs are able to manage and lead with DT in the same way people without MS are able to do.

The finding that DS specifically interferes with walking during DT in pwMSs, as well as in HCs, independently from the difficulty level, suggests a divided attention rather than overall capacity issue. Even if we used the same cognitive domain, it should be noted that Hamilton et al. (10) applied other demand conditions in DS, titrated (calculated to subject's own span at baseline), and fixed, hampering direct comparisons with our findings. Moreover, Hamilton et al. assessed CMI using only one cognitive domain, making it difficult to understand the specific role of task demand. Indeed, using different cognitive domains in the same population was helpful in understanding the mechanism underlying CMI. In our study, CMI on walking was “more severe” as a consequence of the added cognitive task (working memory) and difficulty (the backward one), which would indicate either a bottleneck between tasks (DS and walking) or an overload of brain capacity. These results may be interpreted by theories explaining CMI in humans (40): the capacity-sharing theory, according to which two or more tasks performed together determine an interference when the total available brain capacity is saturated and by the bottleneck theory (40). According to the bottleneck theory, it is the nature of the task (working memory rather than verbal fluency or response inhibition) that determines specific interference, as structural or functional neural pathways may be simultaneously required but not simultaneously used. For more details on these theories, see Leone et al. (40). Another proposed model of divided attention is the one proposed by Kahneman's (41), and it is based around the idea of mental efforts, which is how demanding the processing of a particular input might be. In fact, although some tasks have a high information load, they might be relatively automatic (requiring few demands in terms of mental effort) not leading to a consistent percentage change of gait velocity when a secondary task was added. Moreover, the total available processing capacities may be influenced by other factors such as arousal, enduring dispositions, and momentary intentions, which should be taken into account in future studies. A previous study attempted to evaluate the effect of various cognitive tasks on walking performance (11); however, they examined only one cognitive domain (mental tracking) but changed the difficulty level, and the motor task was to walk on an instrumented walkway, which is considered distinct from over ground walking (42). They found that serially subtracting 7's from 100 resulted in the most consistent changes in walking performance, likely because it was the most difficult task, but there were no significant differences between groups as both pwMSs and HS reduced their velocity.

Another study evaluated the effect of a more demanding motor task, backward waking, compared to forward waking, on walking performance during DT(4). They found that pwMSs walked slower than the HC group during backward walking and even slower when combined with a cognitive task; however, they did not calculate DTCs. Moreover, backward walking cannot be considered a valid measure of real-life walking, as walking while holding a mug is a realistic scenario. Walking while carrying a mug/cup is a complex task with more components than walking alone. However, carrying a mug with the dominant hand is not considered a true secondary task but something similar to the addition of obstacles to the walking path. Indeed, both the cup and the obstacles represent further postural constraints on the system, increasing the task complexity but not changing the number of tasks performed (43). Performing a complex motor task requires a higher attentional load and allocation than simple walking, leading to a higher magnitude of interference than only walking as suggested by the main effect of motor complexity found.

To summarize our findings on gait velocity, (1) working memory tasks are the only cognitive task that reveal the highest CMI leading to a decrease on motor performance in pwMSs and in HCs; (2) CMI induced by a working memory task is independent from a secondary motor task (CWT) added to simple walking. These findings suggest that a long working memory task is able to affect significantly cognitive and motor performance in pwMSs and HCs, whereas all the other cognitive tasks affect less the cognitive–motor performance; pwMSs and HCs are able to do DT at the same “cost.”

### The Effects of 16 Different DT Paradigms on Cognitive Performance: Cognitive DTC

Overall, the results suggest that by3 was the easiest task (highest accuracy), whereas DS_B led to a greater detrimental effect on accuracy (lowest accuracy) in both pwMSs and HCs. Performing a motor CWT has no effect on accuracy changes. The fact that performing DS_B has also the highest detrimental effect over gait velocity suggests this DT combination leads to a true bidirectional interference, at both motor and cognitive levels. Further studies are needed to confirm this hypothesis.

When we analyzed cDTC, the study did not find a significant main effect of groups neither of cognitive task and motor complexity factors. This contrasting results compared with analysis of average accuracy indicates that likely cDTC is not a reliable and sensitive measure of cognitive interference. PwMSs and HCs had a similar pattern of adaptive behavior for DT, likely prioritizing the same task. Moreover, analysis of pooled data (all participants) revealed the mean values of the major part of the cDTCs were negative, suggesting a prioritization of cognitive tasks (cognitive facilitation). Generally, healthy people use a “posture first” strategy in order to avoid hazards and falls. In our study, people were invited to do both tasks as best as they could, without prioritization. The analysis of the percent of change in cognitive task performance from ST to DT revealed that both pwMSs and HCs likely prioritized the cognitive tasks over walking performance, as the accuracy of almost all the tasks was higher during DT compared with ST (sitting). The HC group may have prioritized the cognitive task without significantly reducing walking performance (i.e., gait velocity). The same strategy may also have been adopted by pwMSs, but perhaps to maintain a good cognitive performance, they have to “pay” a higher “cost” by means of a slowing down more. Wadja et al. (44) previously described CMI during walking in MS may result in a cognitive and/or gait interference (detrimental effect) or a cognitive and/or gait facilitation (beneficial effect), as part of a prioritization strategy.

The effect of a motor task on concurrently performed cognitive task while walking (the DTC of cognitive performance) has previously been investigated (10, 17, 18, 45–47). Contrary to our findings, these previous studies have shown that performing a motor task diminished cognitive performance in pwMSs compared to HCs (20, 21). One study in pwMSs showed that the reduction in cognitive performance was related to a higher risk of falling (47). However, many studies did not measure cognitive performance without performing a motor task (no data for single cognitive tasks), making it difficult to verify priority (17, 18).

In contrast to our findings, Hamilton et al. (10) found that pwMSs had greater reductions in cognitive task performance (DS) during DT when compared with HCs. Statistically significant differences were reported in cognitive DTC only during fixed demand DT. Downer et al. (45) showed that the addition of a motor task (walking) to a mental tracking task (subtracting sevens) worsened performance by >50% in pwMSs but not in HCs. We used both the above cognitive distractors but did not find significant changes in cognitive performances from ST to DT. These conflicting results may be due to the fact that Hamilton et al. used (1) an instrumented walkway for the walking trials, and (2) a fixed version of DS with a slight difference in DS duration (90 vs. 60 s in our study). Regarding comparison to Downer et al., it is difficult to draw conclusions, as they enrolled fewer participants without reporting power size calculation; furthermore, they used a different methodology: (1) our participants walked on ground being monitored by APDM wearable sensors, whereas in the study of Downer et al. (45), subjects walked on a instrumented walkway; (2) our participants walked along an 8-m corridor for a fixed time (15 s), whereas in Downer et al. (45), they had to walk 16 m (without a time restriction). Moreover, it should be noted that, in our study, the accuracy in subtracting by seven tasks while in a sitting position (ST) was very similar between groups, whereas in Downer et al. (45), there was an non-significant trend toward a difference between groups (with pwMSs scoring better than HCs); therefore it could be argued that pwMSs had a higher cDTC as they started from a “random” higher score. We have data on simultaneous walking performance, whereas they did not measure mDTC or motor changes from ST to DT. Further studies are necessary to confirm our results.

### Patients' Physical or Cognitive Baseline Characteristics as Potential Contributing Factors for DTC

As previously reported in pwMSs with moderate disability, our results confirm a decreased baseline walking velocity (walking in ST) in comparison with HCs. Sosnoff et al. (14) found differences in all gait parameters during ST between patients with mild, moderate, and severe disability, but the differences were less when DTC, which was taken into account, suggesting that DT when walking evaluates an aspect other than the motor assessment itself. On the other hand, Learmonth et al. (37) reported that there were no significant differences in gait parameters during DT or DTC between pwMSs with different disability levels and HCs.

Consistent with the results of Sosnoff et al. (14), our results suggest that disability level impacts the magnitude of mDTC. We found a significant correlation between mDTC and motor measures collected as baseline physical characteristics (T25WT, 6MWT, and EDSS). However, the disability level measured by EDSS was the only independent variable with a significant effect on mDTC.

There are several possible reasons why pwMSs with more severe disabilities showed a larger DTC. One could be that cognitive function declines with disease progression, as indicated by disability in pwMSs (48, 49); therefore, it is possible that the additive cognitive task resulted in greater dysfunction in pwMSs with lower information processing speed capacity. Moreover, in participants with motor disability, a longer task (such as DS) could lead them to exceed their limited exertional capacity leading to a greater DTC. Our results contradict those reported by Hamilton et al. (10), who did not find a relationship between DTC during walking and EDSS. However, they had small sample size, and cane use was an exclusion criterion.

Regarding the role of baseline cognitive status, we found that almost 30% of our participants with MS had CI, whereas none of our HCs had CI.

All together, these findings suggest that pwMSs with higher motor disability and lower information processing speed may be those who perform worse in certain DT paradigms. Also, Hamilton et al. found significant associations between baseline cognition variables and DTC. They found a significant association between general cognitive functioning and DTC when walking, with cognitive function assessed by ACE-R, a brief cognitive test designed to screen for dementia, which is not validated for MS.

Motl et al. (50) reported that both walking performance and cognitive processing speed (measured by the symbol digit modality test) correlated with walking DTC. Moreover, Sandroff et al. (38) demonstrated a significant relationship between CMI and inhibitory control processes in HCs but not in pwMSs (38). Other cognitive functions, such as divided/alternating attention, response inhibition, set shifting, and working memory, were considered particularly relevant to DT walking (51). Interestingly, we found a significant correlation between mDTC and the Trail Making Test. The Trail Making Test is a measure of attention and executive control (52), as it explores the ability to maintain a cognitive set (53) as well as alternating attention.

## Limitations

Our study has some limitations, such as the small sample size and the study heterogeneity of the study population, given to explorative nature of the study. The small sample size halted us to perform different statistical analysis, as well as to compare DTC between subgroups (i.e., disability level, cognitive status). We did not collect data on fatigue, pain, medications, and other MS-specific variables, which we suggest to measure in future studies in order to explain better CMI in such a disease.

## Research and Clinical Implications

Our study may have clinical and research implications. Clinically, it may increase the awareness as to the importance of testing DT when walking, taking into account the type of cognitive task chosen and/or adding of another motor task. As different types of cognitive tasks may result in different patterns of CMI, the specific type of cognitive activity may be used for DT rehabilitation of walking. On the other hand, DT rehabilitation strategies may be targeted toward the modulation of walking conditions in order to allocate higher attentional resources for better performance on a complex cognitive task.

Further research is needed in order to assess the specificity and sensitivity of our results, as well as the mDTC profile in pwMSs.

Another point to be further investigated is the role of longer tasks (60″ rather than 15″) on motor interference in pwMSs; future studies investigating the role of walking-related motor fatigue and mental fatiguing are warranted.

We recommend to use mDTC of DS_B as a useful outcome measure to detect ecologically valid DT impairments, but more research is first required.

## Conclusion

The present study presented eight different cognitive tasks with varying degrees of difficulty during walking alone or during CWT. Our finding that no specific combination of walking while doing a second motor task was able to discriminate pwMSs from HCs led to the conclusion that pwMSs and HCs behave in the same manner independently from the cognitive task and the motor complexity of the DT combinations applied. We demonstrate that the overall magnitude of the CMI depends on the type and complexity of the additional task.

## Data Availability Statement

The raw data supporting the conclusions of this article will be made available by the authors, without undue reservation.

## Ethics Statement

The studies involving human participants were reviewed and approved by Ethics Committee University Hasselt. The patients/participants provided their written informed consent to participate in this study.

## Author Contributions

CL, FP, and PF contributed conception and design of the study. IB, RV, and LM organized the database. CL and LM performed the statistical analysis. CL wrote the first draft of the manuscript. LM wrote the statistical analysis section of the manuscript. EV wrote part of methods and results section. BV, LM, RV, and PF wrote sections of the manuscript. All authors contributed to manuscript revision and read and approved the submitted version.

## Conflict of Interest

The authors declare that the research was conducted in the absence of any commercial or financial relationships that could be construed as a potential conflict of interest.
